# COVID-19 Pandemic Adversely Affects the Provision of Desired Newborn Circumcision: Perinatal Physician Perspectives

**DOI:** 10.3389/frhs.2021.799647

**Published:** 2022-01-17

**Authors:** Lauren E. Corona, Ilina Rosoklija, Ryan F. Walton, Derek J. Matoka, Catherine M. Seager, Jane L. Holl, Emilie K. Johnson

**Affiliations:** ^1^Division of Urology, Department of Surgery, Ann and Robert H. Lurie Children's Hospital of Chicago, Chicago, IL, United States; ^2^Department of Urology, Northwestern Feinberg School of Medicine, Chicago, IL, United States; ^3^Department of Neurology, University of Chicago, Chicago, IL, United States

**Keywords:** circumcision, perinatal care, newborn circumcision, COVID-19 pandemic, neonate, clamp circumcision, pandemic

## Abstract

Over half of boys in the United States undergo circumcision, which has its greatest health benefits and lowest risks when performed during the newborn period under local anesthesia. The COVID-19 pandemic has affected delivery of patient care in many ways and likely also influenced the provision of newborn circumcisions. Prior to the pandemic, we planned to conduct a qualitative study to ascertain physician perspectives on providing newborn circumcision care. The interviews incidentally coincided with the onset of the pandemic and thus, pandemic-related changes emerged as a theme. We elected to analyze this theme in greater detail. Semi-structured interviews were conducted with perinatal physicians in a large urban city from 4/2020 to 7/2020. Physicians that perform or counsel regarding newborn circumcision and physicians with knowledge of or responsibility for hospital policies were eligible. Interviews were transcribed verbatim and qualitative coding was performed. Twenty-three physicians from 11 local hospitals participated. Despite no specific COVID-19 related questions in the interview guide, nearly half of physicians identified that the pandemic affected delivery of newborn circumcision care with 8 pandemic-related sub-themes. The commonest sub-themes included COVID-19 related changes in: (1) workflow processes, (2) staffing and availability of circumcision proceduralists, and (3) procedural settings. In summary, this qualitative study revealed unanticipated COVID-19 pandemic-related changes with primarily adverse effects on the provision of desired newborn circumcisions. Some of these changes may become permanent resulting in broad implications for policy makers that will likely need to adapt and redesign the processes and systems for the delivery of newborn circumcision care.

## Introduction

Over half of boys in the United States undergo circumcision (1), a procedure that has its greatest health benefits and lowest risks when performed during the newborn period under local anesthesia (2). The COVID-19 pandemic has affected delivery of patient care in many ways and likely has also influenced the provision of newborn circumcisions. Prior to the pandemic, we planned to conduct a qualitative study to ascertain general physician perspectives on providing newborn circumcision care. The interviews incidentally coincided with the onset of the pandemic and thus, COVID-19 pandemic-related changes emerged as a theme. The aim of the present study was to analyze this theme in greater detail and to examine physician perspectives on the effect of the COVID-19 pandemic on the provision of newborn circumcision care.

## Methods

Convenience sampling was employed for participant recruitment via electronic mail to physicians at Chicago Area birthing hospitals. A secondary snowball sampling approach was then used to ensure broad representation of hospital location (city vs. suburbs), size, type (public vs. private), socioeconomic patient characteristics, and specialty performing circumcisions. Physicians of any specialty that perform newborn circumcision and/or counseling regarding circumcision and physicians with knowledge of or responsibility for hospital policies and practices were eligible. Recruitment concluded when preliminary analysis of interviews revealed saturation of themes.

Semi-structured interviews were conducted with perinatal physicians in Greater Chicago by one investigator (EKJ) via videoconference or telephone from 15 April to 13 July 2020. The interview guide, which was developed by the study team of general pediatricians, pediatric urologists, and health services and outcomes researchers, included the following general topics: patient demographics, clinician comfort/experience with circumcision, discussions with families, process and procedure details, and barriers and facilitators to circumcision care (Supplement). The interview guide was tested and revised with 2 uninvolved individuals prior to implementation with participants and iteratively updated after the first several interviews. Study participants completed a brief survey about their demographics and hospital characteristics. Interviews were audio-recorded and transcribed verbatim.

Qualitative coding was performed via an inductive and deductive approach within a content analysis framework, facilitated by the MAXQDA qualitative analysis software. Line-by-line coding was independently performed by three authors (IR, JLH, and EKJ) to create focused codes. Each discrepancy was reviewed and verbally discussed until consensus was achieved. Focused codes were then summarized into higher level themes. When COVID-19 pandemic-related changes emerged as a prevalent theme, one author (LEC) independently performed line-by-line coding to further deconstruct this theme into sub-themes. Representative quotations were then discussed and chosen, ensuring that a broad representation of participant perspectives was included. Descriptive statistics were used to summarize survey data. The study was reviewed and approved by the Ann and Robert H. Lurie Children's Institutional Review Board.

## Results

Twenty-three perinatal physicians (10 family medicine, 8 pediatricians, and 5 obstetricians) from 11 local hospitals participated. Study participant demographics are shown in Table 1. Despite no specific COVID-19-related questions in the interview guide, nearly half (10/23, 44%) of physicians identified that the COVID-19 pandemic affected delivery of newborn circumcision care. Participants described these changes in response to a variety of questions including descriptions of their procedural experience and comfort, procedural logistics, patient demographics, discussions with families, and barriers to newborn circumcision. Responses were classified into eight pandemic-related sub-themes (Table 2).

**Table 1:** Study participant demographics.

	**Total (*n* = 23)**
**Gender No. (%)**	
Male	5 (22%)
Female	18 (78%)
**Spanish/hispanic/latino origin No. (%)**	
Yes	1 (4%)
No	22 (96%)
**Race No. (%)**	
White	17 (74%)
Black or African American	3 (13%)
Asian	1 (4%)
Other	2 (9%)
**Specialty No. (%)**	
Family medicine	10 (43%)
Pediatrics	8 (35%)
Obstetrics & gynecology	5 (22%)
**Years since graduating medical school**	
Minimum	5.0
Maximum	38.0
Mean	19.2
**Primary institution No. (%)**	
A	5 (22%)
B	3 (13%)
C	3 (13%)
D	3 (13%)
E	3 (13%)
F	1 (4%)
G	1 (4%)
H	1 (4%)
I	1 (4%)
J	1 (4%)
K	1 (4%)
**Educational leadership role No. (%)**	
Yes	10 (44%)
No	13 (57%)
**Other hospital leadership role No. (%)**	
Yes	12 (52%)
No	11 (48%)
**Currently performing circumcisions No. (%)**	
Yes	18 (78%)
No	5 (22%)
**Estimated # of personal circumcisions per year**	
Minimum	0.0
Maximum	250.0
Mean	56.6
**Employed at minority predominate institution No. (%)**	
Yes	18 (78%)
No	5 (22%)
**Employed at institution with perceived health literacy challenge No. (%)**
Yes	19 (83%)
No	4 (17%)

**Table 2:** Sub-themes related to theme of changes in newborn circumcision care secondary to the COVID-19 pandemic.

**Sub-theme**	**Exemplar quote**
Delays secondary to changes in workflow processes	“I think, you know, everyone who is designated to be doing the circs [during COVID] are also designated to be covering actively laboring patients…so, obviously, actively laboring or OB triage patients have to come first. So, sometimes the timing [of circumcision] gets pushed off.” *Participant 21*
Changes in staffing/proceduralist availability	“…now that COVID is going on, um, our family medicine has been pulled out to be more in the clinic. So, the OBs are doing all of them.” *Participant 15*
Changes in procedural settings	“…since the pandemic, we haven't been doing them in the office.” *Participant 16*
Changes in procedural logistics/support	“I tell them if they want the circumcision done, they're going to have to wait until the baby is at least 24 h and also during a reasonable time because I'm not going to do circumcisions at like 6 P.M.” *Participant 10*
Provider practice changes	“And then in the outpatient setting, I have two clinics a week, which-now one of them is just telemedicine because of COVID.” *Participant 23*
Changes in parental counseling	“So, they were like, ‘no, you don't need it. It's elective.'” *Participant 15*
Acceleration of previously planned changes to systems^a^	“We had planned to change to inpatient in May anyway…so COVID came and we just switched early.” *Participant 05*
Changes in patient demographics	“…and especially during COVID, it's been a much larger percentage of Hispanic patients.” *Participant 21*

a *This was the only positive change described in all the themes related to COVID-19*.

One prevalent sub-theme was delays secondary to changes in workflow processes. For example, early discharge during COVID-19 discouraged families from waiting for an available provider to perform circumcision resulting in deferment of the procedure. In addition, responsibilities that had previously been covered by multiple providers were now being covered by a single provider. One participant (ID 21) noted: “*I think, you know, everyone who is designated to be doing the circs [during COVID] are also designated to be covering actively laboring patients…so, obviously, actively laboring or OB triage patients have to come first.”*

A second prevalent sub-theme was the pandemic-related changes in staffing and availability of circumcision proceduralists. For example, to minimize personnel in house, one hospital limited the newborn nursery rounders to be pediatricians that were also capable of performing newborn circumcision. Another hospital that previously had family medicine physicians come in from the outside to perform circumcisions limited the circumcisers during COVID-19 to the obstetricians that were covering labor and delivery. As noted by one participant (ID 15): “*…now that COVID is going on, um, our family medicine has been pulled out to be more in the clinic. So, the OBs are doing all of them.”*

COVID-19-related changes to the procedural setting was another frequent sub-theme. For example, one participant (ID 16) described the closure of outpatient circumcision clinics and a shift to the exclusive performance of circumcisions in the inpatient setting: “*…since the pandemic, we haven't been doing them in the office.”* In contrast, others described new trends of early discharge resulting in an increased need for outpatient circumcisions.

While the majority of participants described negative changes, delays, or barriers secondary to the pandemic, one individual described a positive change (acceleration of a previously planned systems change to move all circumcisions to the inpatient setting). Other transitions that were described included changes in procedural logistics/support, provider practices, in the counseling of parents, and in patient demographics. Examples include shortages of equipment and nurses, a need for emphasis in counseling of parents on the elective status of circumcision, less time allocation devoted to clinic procedures, and an increase in numbers of Hispanic patients seen during the pandemic.

## Discussion

Physicians, who were being interviewed for a separate study about disparities in access to desired newborn circumcision at the beginning of the COVID-19 pandemic, identified, without being asked specifically, several pandemic-specific barriers to performing newborn circumcision. This was an unanticipated, albeit not surprising finding. To our knowledge, the effect of the pandemic on newborn circumcision has not been previously reported, although it is well-known that the pandemic affected nearly all aspects of healthcare delivery (e.g., suspension of elective surgery, telemedicine).

The need to optimize hospital resources has been a common pandemic-related theme that was also reported by participants about newborn circumcision. Many physicians noted the shift in newborn circumcision from the inpatient to outpatient setting, driven in part by early discharge in order to optimize bed utilization. Indeed, many previously inpatient-only procedures shifted to the outpatient setting with good results (3). Similarly, the desire to reduce the number of providers in the hospital resulted in fewer physicians being available to perform newborn circumcisions. Staffing availability, as hospitals redeployed staff to COVID units, had a down-stream impact on availability for assistance with newborn circumcisions.

Our analysis found a majority of negative pandemic-related changes. For example, one physician indicated that some families that desired circumcision would delay discharge solely for this indication since hospital policy required a minimum of 24 h waiting period for inpatient circumcision: a poor utilization of hospital resources during a time where they are limited. However, a pandemic-related facilitator to newborn circumcision was also described. One provider noted earlier implementation of a planned shift to all circumcisions being performed inpatient, resulting in easier access to desired newborn circumcision for families. Recognizing these changes, both positive and negative, will allow us to reevaluate indications for current practices and revise as appropriate.

The COVID-19 pandemic was a sudden crisis that affected all healthcare systems across the world necessitating the rapid implementation of non-evidence based procedures and policies. These changes were inconsistent nationally and varied amongst hospitals within even the same city. A recent nationwide survey study by Aragona et al. found that for COVID-19-positive mothers, 38% of surveyed institutions deferred newborn circumcision. There were additional high, but inconsistent modification rates to routine newborn screening, discharge, and follow-up plans (4). With increasing evidence and experience as the pandemic persists, the policies and practices surrounding newborn circumcision care continue to evolve. Currently, evidence is unavailable but anecdotally, many of these practice and proceduralist changes have been sustained. Future research should attempt to evaluate these trends and their effect on costs, newborn and maternal outcomes, and childhood anesthetic exposure. These results likely have broad implications for many elective procedures and this information should be used to guide care redesign and further policy making.

This study does have limitations. It was conducted in a single United Sates city and results might not be generalizable to the entire population. However, it captured perspectives from 23 perinatal physicians from a variety of specialties from 11 different institutions. Chicago is the third largest city in the United States, and the birthing hospitals in the greater Chicago area have been shown to represent a racially/ethnically and sociodemographically diverse population (5). In addition, this study was designed to examine disparities in access to desired newborn circumcision and it was coincidental that the interviews were conducted at the beginning of the pandemic resulting in pandemic-related changes to emerge as a theme. Therefore, we did not collect any COVID-19-specific hospital level data. In addition, given that the temporality of these results is limited to the early months of the pandemic, further research will be needed to explore which of these changes have persisted.

## Conclusions

In summary, this qualitative study revealed unanticipated COVID-19 pandemic-related changes with primarily adverse effects on the provision of desired newborn circumcisions. Some of these changes may become permanent resulting in broad implications for policy makers that will likely need to adapt and redesign the processes and systems for the delivery of newborn circumcision care.

## Data Availability Statement

The raw data supporting the conclusions of this article will be made available by the authors, without undue reservation.

## Author Contributions

LC conceptualized and designed the study, drafted the initial manuscript, and approved the final manuscript as submitted. EJ conceptualized and designed the study, conducted participant interviews, critically reviewed the manuscript, and approved the final manuscript as submitted. IR conceptualized and designed the study, critically reviewed the manuscript, and approved the final manuscript as submitted. RW, DM, CS, and JH critically reviewed the design of the study, critically reviewed the manuscript, and approved the final manuscript as submitted. All authors contributed to the article and approved the submitted version.

## Conflict of Interest

The authors declare that the research was conducted in the absence of any commercial or financial relationships that could be construed as a potential conflict of interest.

## Publisher's Note

All claims expressed in this article are solely those of the authors and do not necessarily represent those of their affiliated organizations, or those of the publisher, the editors and the reviewers. Any product that may be evaluated in this article, or claim that may be made by its manufacturer, is not guaranteed or endorsed by the publisher.
