# Supplementary material for: COVID-19 Pandemic Adversely Affects the Provision of Desired Newborn Circumcision: Perinatal Physician Perspectives
Source: Front Health Serv. 2022 Jan 17;1:799647. doi: 10.3389/frhs.2021.799647 (PMC10012608; doi:10.3389/frhs.2021.799647)
Supplement: Supplementary file 1 [file Table_1.DOCX]

**Supplement**

**Physician Interview Guide**

1. **Introduction: (5 Minutes)**

Thank you for participating. My name is [*your name*] and I am a [*medical student/fellow/research coordinator*] working with the Division of Pediatric Urology at Ann & Robert H. Lurie Children’s Hospital of Chicago. We are currently investigating neonatal circumcision practices in the Chicago area. We have asked you to take part in this study because your specialty is one that typically performs or has knowledge about neonatal circumcisions in some capacity. Today, I’d like to hear about your clinical practices, experiences, and expertise regarding circumcision of newborn boys.

Before I begin, I want us both on the same page about how this discussion will proceed.

1. This discussion is confidential.
2. The questions have no right or wrong answer.
3. My primary goal in this conversation is gain an understanding of your perspective on how patients who are born at your hospital obtain neonatal circumcision. We’ll also talk a bit about your background, the patient population you treat, and your thoughts in general about routine neonatal circumcision
4. Please feel free to interject any thoughts, opinions, or experiences you have regarding this subject, even if it is beyond the scope of or secondary to the question.
5. After we are finished, I will ask you to fill out a brief survey.

Audiotapes:

I want to let you know that I am audio-taping the discussion so that we can accurately record what you have said, and then transcribe it for analysis on a later date. Only the research staff will listen to this audio tape.

Questions or concerns: Do you have any questions or concerns at this point before we begin?

1. **INTERVIEWEE PROFESSIONAL EXPERIENCE (3-5 minutes)**

Please tell me about your role as an attending physician at your main practice site.

1. What leadership positions or other titles do you have?
2. What is your role in performing neonatal circumcisions at your main practice site?
3. Is there anything else related to your role at the hospital that may be relevant to neonatal circumcisions?
4. **PATIENT DEMOGRAPHICS (5-8 minutes)**

Ok, let’s talk a bit about the patients at your main practice site. I’d like to get a sense of the demographics of the families you treat.

- 1. How would you describe the socioeconomic status of the families you treat?
  2. What do you know about the insurance status of the families you treat?
     - 1. Do you know how many are:
          - privately insured
          - publicly insured
          - uninsured
  3. What do you know about the education levels of the families you treat?
     - 1. Did most parents graduate from high school, college?
       2. How frequently do you perceive health literacy to be a challenge for your families?
  4. How would you describe the racial and ethnic background of the families you treat?
     - 1. What proportion would you say are
          - White-Non-Hispanic
          - African American-Non-Hispanic
          - Hispanic
          - Asian
          - Native American/Alaskan Native
          - Native Hawaiian/Pacific Islander
          - Mixed Race
  5. What is the English-speaking ability of the families you treat?
     - 1. To your knowledge, what percentage of families require a family member or medical interpreter to communicate in a healthcare setting?
  6. Is there anything unique about the demographics of the families you treat that I did not address but you think is relevant?

1. **NEONATAL CIRCUMCISION - TRAINING AND COMFORT (5 minutes)**

Now, let’s talk for a few minutes about your training and level of comfort performing neonatal circumcisions.

- 1. Describe how you learned to perform neonatal circumcisions.
  2. What formal instruction or training on how to perform neonatal circumcisions have you received?
     - 1. If so, what was the training like?
       2. When did you receive it?
       3. Where did you receive the training?
       4. Were you a trainee or an attending when receiving training?
  3. What additional training do you think would be most helpful for physicians performing neonatal circumcisions?
     - 1. What immediate training needs does your hospital have?
  4. Please describe your current level of comfort performing neonatal circumcision
     - 1. What could be done to increase your level of comfort?
  5. How many neonatal circumcisions do you personally perform in a year?
     - 1. If zero, why don’t you perform neonatal circumcisions?
  6. How frequently do you refer families to another clinician for neonatal circumcision?
     - 1. In what situations do you refer?
       2. To whom do you typically refer families?
     1. Any other comments about neonatal circumcision training or comfort

**V.** **NEONATAL CIRCUMCISION – DISCUSSIONS WITH FAMILIES (10-15 minutes)**

I’d now like to focus on some questions related your experience discussing and offering neonatal circumcision.

1. What is your general opinion of routine neonatal circumcision?
   1. What ethical concerns do you have about offering the procedure?
2. Describe your clinical approach regarding discussing offering neonatal circumcision
   1. How does the topic of neonatal circumcisions typically come about?
   2. Do the parents or health care team typically initiate the conversation?
   3. What healthcare team members usually discusses circumcision with parents?
   4. How does a typical discussion go?
   5. What types of questions do families ask?
   6. How do you discuss the risk and benefits of neonatal circumcision with families?
3. What estimated proportion of families at your hospital ultimately decide for a routine neonatal circumcision?
4. What types of educational tools could be helpful in discussing neonatal circumcision with families?
   1. Does your hospital currently provide any such tools?
5. What has been your experience of seeing and hearing about neonatal circumcision complications?
   1. (If applicable – how have these experiences changed what you tell families?)
6. Any other comments related to discussing neonatal circumcision with families that you’d like to share?

**VI. NEONATAL CIRCUMCISION - PROCEDURE (10 minutes)**

Assuming parents have chosen for their son to undergo routine neonatal circumcision, I’d like to get a sense of the process by which this happens. This is the part of our conversation where I want to be especially sure to understand all the details, so please tell me everything you think might be relevant.

1. What are families typically asked to do before birth if they might want a neonatal circumcision?
2. Let’s say a family with a newborn their son to be circumcised, please describe what happens next.
   1. Please walk me through the steps of what happens from request through the time a baby has the circumcision.
   2. Who do they need to tell that they want the circumcision?
   3. How is payment handled?
3. *(If not covered already): Tell more me about the setting and circumstances in which neonatal circumcisions are performed.*
   1. *Are they typically performed in the inpatient or outpatient setting?*
      1. *Do you know why? (system, coverage, billing, hospital policy, reimbursement, etc.)*
      2. *If inpatient, how does the procedure impact the neonate’s hospital stay?*
   2. *What provider type and specialty usually performs the procedure?*
   3. *What is done for pain control and soothing?*
   4. *What type of device is used?*
4. *(If not covered already): How resource intensive is the procedure?*
   1. *How many clinicians are involved?*
   2. *How long does the procedure take?*
5. What important aspects of the procedure have we not yet covered?
6. **CIRCUMCISION – BARRIERS AND FACILITATORS (5 minutes)**

Finally, I’d like to wrap up by asking for your opinion about barriers to and facilitators of neonatal circumcision.

1. In your opinion, what barriers prevent boys at your hospital from receiving desired neonatal circumcisions?
   1. Examples to prompt: financial, time constraints, provider training, provider availability, parental concerns/difficulty deciding?
   2. Can you think of anything that can be done to address the barriers you’ve identified (if there are any)?
2. What works well regarding offering and performing neonatal circumcisions at your hospital?
   1. Can you offer any “best practices” for other hospitals to consider?
3. Do you know if your hospital has any specific policies regarding neonatal circumcision? (Yes/No)
   1. If so, can you describe the policy to me?
   2. If so, do you agree with this policy?
      1. Why or Why not?
4. Are there any additional team members that you recommend we speak with at your hospital that have insight into the clinical or administrative (policy) aspects of offering and performing neonatal circumcision?
   1. Any non-physicians, and/or hospital administrators?
5. Which other area hospitals should we contact who may share a different perspective?
   1. Would you be willing to share specific name/contact information, or make an introduction if you have a recommended contact?

That’s all for the planned questions I have - is there any additional insight about neonatal circumcision you would like to share before we wrap up?

**I’m pasting a link to the 1 minute survey in the chat box now. Your study ID is _______.**

Thank you for your time.
